# Duplex Reverse-Transcription Real-Time Polymerase Chain Reaction Assay Targeting 23S rRNA Single Nucleotide Polymorphisms for the Detection of Flea-Borne Rickettsioses

**DOI:** 10.4269/ajtmh.23-0884

**Published:** 2024-07-16

**Authors:** William S. Probert, Alexa C. Quintana, Anne M. Kjemtrup, Jill K. Hacker

**Affiliations:** ^1^ Viral and Rickettsial Disease Laboratory, California Department of Public Health, Richmond, California;; ^2^ Infectious Diseases Branch, Vector-borne Disease Section, California Department of Public Health, Sacramento, California

## Abstract

Flea-borne spotted fever and flea-borne (murine) typhus are rickettsioses caused by *Rickettsia felis* and *Rickettsia typhi*, respectively, and typically present as undifferentiated febrile illnesses. The relative contribution of these agents to flea-borne rickettsioses in California is unclear. We have developed a duplex reverse transcription real-time polymerase chain reaction (RT-rtPCR) assay targeting *R. felis*– and *R. typhi*–specific 23S ribosomal RNA single nucleotide polymorphisms to better understand the respective roles of these agents in causing flea-borne rickettsioses in California. This assay was compared with an established duplex *R. felis*– and *R. typhi–ompB* rt-PCR assay and was shown to have 1,000-fold and 10-fold greater analytical sensitivity for the detection of *R. felis* and *R. typhi*, respectively. Retrospective testing of clinical specimens with both assays established *R. typhi* as the major etiologic agent of flea-borne rickettsioses in California.

## INTRODUCTION

Rickettsioses are acute febrile illnesses caused by small, obligate intracellular, gram-negative bacteria belonging to the genus *Rickettsia*. Infected ticks, fleas, mites, and lice serve as vectors for disease transmission. The flea-borne rickettsioses include flea-borne spotted fever (FBSF) and flea-borne typhus (FBT; also known as murine or endemic typhus) caused by *Rickettsia felis* and *Rickettsia typhi*, respectively. The clinical features of FBSF and FBT are similar and typically include nonspecific symptoms such as fever, headache, and myalgia, although FBSF is associated with milder disease than FBT.^1^ A papular to maculopapular rash is associated with most FBSF patients but less frequently observed with FBT patients. Severe clinical manifestations are rare but may include pneumonitis, renal injury, and central nervous system involvement. Therapeutic intervention with a tetracycline class of antibiotic such as doxycycline is highly effective in the management and recovery of patients with flea-borne rickettsioses.

Both *R. felis* and *R. typhi* are distributed worldwide. Flea-borne spotted fever and FBT are not nationally notifiable diseases in the United States; however, reporting of suspected FBT cases is required by some local and state public health departments. The majority of FBT cases in the United States are reported from Texas and California, with 580 and 216 cases, respectively, recorded in 2022.^2^^,^^3^ Endemic foci of FBT are maintained through an urban cycle of *R. typhi* transmission, involving rats as the reservoir host and rat fleas as the vector, and a suburban cycle in which opossums serve as the reservoir and cat fleas as the vector.^4^^,^^5^ Recent ecological epidemiology investigations have illustrated the relative importance of the suburban cycle for *R. typhi* maintenance and transmission in Texas and California.^6^^,^^7^

Since the initial description of an *R. felis* infection in a patient from Texas, additional cases of FBSF have not been recognized in the United States.^1^ In contrast, *R. felis* infections appear to be relatively common in sub-Saharan Africa and Asia; however, reports of *R. felis* detections in both healthy and febrile individuals in Africa have brought into question the true incidence of FBSF in this region and the pathogenic potential of *R. felis*.^1^^,^^8^ The invertebrate reservoir host and vector for *R. felis* is the cat flea, *Ctenocephalides felis*.^8^ Surveys of cat fleas have shown that *R. felis* infections are far more prevalent than *R. typhi* infections, suggesting that the incidence of FBSF should be much greater than the incidence of FBT in California.^7^^,^^9^ These observations suggest that limited awareness of FBSF by clinicians and lack of available diagnostic tests may lead to underestimation of human *R. felis* infections. Alternatively, *R. felis* may have limited infectivity or minimal pathogenic potential for humans.

Serologic detection using indirect immunofluorescence has been the gold standard for laboratory confirmation of rickettsioses, providing group- but not species-level identification. The usefulness of serologic confirmatory testing is also limited by the need to demonstrate seroconversion or a 4-fold rise in titer for paired sera, hindering its timeliness for laboratory diagnosis.^10^ Molecular detection of *Rickettsia* nucleic acids offers a rapid genus-, group-, or species-specific alternative to serologic testing.^11^ Recently, a Pan-*Rickettsia* reverse transcription real-time polymerase chain reaction (RT-rtPCR) assay was described that targets 23S ribosomal RNA (rRNA), a ribosomal component that is present at high copy numbers within each bacterial cell.^12^ With this assay, the relatively abundant 23S rRNA is reverse transcribed, after which the desired target is amplified with specific primers and detected with a specific fluorogenic hybridization probe. The RT-rtPCR assay proved to have superior analytical sensitivity when compared with an earlier Pan-*Rickettsia* 50S ribosomal protein L16 (single-copy DNA target) rtPCR assay.

We have expanded upon this work by developing a duplex RT-rtPCR assay targeting 23S rRNA single nucleotide polymorphisms (SNPs) for the detection and discrimination of *R. felis* and *R. typhi* in specimens from suspected flea-borne rickettsiosis cases. We describe here the performance characteristics of this new RT-rtPCR assay measured against an established duplex *ompB* rtPCR assay.^13^ Retrospective testing of 87 clinical specimens revealed the relative contributions of *R. felis* and *R. typhi* as the cause of flea-borne rickettsioses in California.

## MATERIALS AND METHODS

### Nucleic acids.

Nucleic acids from Rickettsiales cell culture isolates were obtained from the Rickettsial Zoonoses Branch, U.S. Centers for Disease Control and Prevention, and BEI Resources (Manassas, VA) (Supplemental Table 1). The concentrations of *R. felis* and *R. typhi* nucleic acids were determined by quantitative PCR using the Pan-*Rickettsia* RCKr assay,^12^ absent the reverse transcription step, and a standard curve generated with quantified plasmid DNA; concentrations were expressed as genome copies per microliter. Nucleic acids from non-rickettsial pathogens associated with fever and rash illnesses were obtained from the California Department of Public Health (CDPH) strain and specimen collections (Supplemental Table 1). In addition, nucleic acids extracted from 10 individual *R. felis*–infected cat fleas (*C. felis*) collected within Orange County, CA, were provided by the Orange County Mosquito and Vector Control District (Garden Grove, CA).

### Clinical and contrived specimens.

A total of 117 samples were used to assess assay performance. These included human clinical specimens (81 sera, 4 plasmas, and 2 whole bloods) collected between April 2017 and January 2023 from 87 case-patients that were submitted to the CDPH Viral and Rickettsial Disease Laboratory for confirmatory testing and 30 *R. felis*–contrived specimens. Specimens were collected for public health surveillance and were considered exempt from human subject regulations by the California Health and Human Services Agency Committee for the Protection of Human Subjects (Project #2023-085). Total nucleic acids were extracted from 300 µL of clinical specimen with the NucliSENS easyMAG instrument (bioMerieux, Durham, NC) and eluted in a final volume of 110 µL. To address the expected paucity of *R. felis* detections, contrived specimens were prepared by spiking *Rickettsia*-negative nucleic acid extracts from 30 serum specimens with nucleic acids from *R. felis*–infected fleas. To do so, nucleic acid extracts from 10 individual *R. felis*–infected fleas were diluted 1:100, 1:1,000, and 1:10,000 and combined with *Rickettsia*-negative nucleic acids at a ratio of 1:9.

### Duplex *R. felis*/*R. typhi* 23S rRNA SNP RT-rtPCR assay.

*Rickettsia felis* and *R. typhi* species-specific SNPs were identified through BLAST searches of the National Center for Biotechnology Information (NCBI) nr/nt and whole-genome shotgun contig databases using the complete 23S rRNA sequence for *R. felis* strain URRWXCal2 (GenBank Accession NR_076359.1) or *R. typhi* strain Wilmington (GenBank Accession NR_076209.1) and multiple sequence alignment of 35 *Rickettsia* species and subspecies with validly published names (Supplemental Table 2).^14^ Species-specific SNPs were confirmed for all available *R. felis* or *R. typhi* 23S rRNA sequences in the NCBI nr/nt database.

The primer and probe sequences for the duplex RT-rtPCR assay were designed using RealTimeDesign SNP genotyping software (Biosearch Technologies, Novato, CA) (Table 1). The RT-rtPCR mixture consisted of 1× One Step PrimeScript III RT-PCR master mix (Takara Bio USA, San Jose, CA), the Rfel23S_F and Rfel23S_R primers at 200 nM, the Rfel23S_P probe at 100 nM, the Rtyp23S_F and Rtyp23S_R primers at 400 nM, and the Rtyp23S_P probe at 300 nM. The nucleic acid input volume was 5 µL for a final reaction volume of 25 µL. Reverse transcription, amplification, and fluorescence detection were performed using an ABI 7500 FAST DX Sequence Detection System (Thermo Fisher Scientific, Carlsbad, CA) with the following cycling parameters: 53°C for 10 minutes, 95°C for 2 minutes followed by 45 cycles of 95°C for 3 seconds and 57°C for 40 seconds. Fluorescent readings were collected during the 57°C anneal/extension step.

**Table 1:** *Rickettsia felis*/*Rickettsia typhi* duplex reverse transcription real-time PCR oligonucleotide primers and probes

Assay Analyte	Oligonucleotide Name	Reference Sequence Coordinates	Oligonucleotide Sequence and Modifications*	Assay Oligonucleotide Concentration
*R. felis* 23S rRNA	Rfel23S_F	NR_076359.1: 1302–1321	GTCCAAGGGTTCTTGCGTAA	200 nM
	Rfel23S_R	NR_076359.1: 1348–1369	GCCTTTCAGCCTCATCTTAGGA	200 nM
	Rfel23S_P	NR_076359.1: 1322–1343	ABY-AGTTAATCTGCACAAGGTTAGT-QSY	100 nM
*R. typhi* 23S rRNA	Rtyp23S_F	NR_076209.1: 1929–1952	GAAAGACCCCGTGAACCTTTACTA	400 nM
	Rtyp23S_R	NR_076209.1: 2001–2020	CTAACGCCTCTGCTTCGCAG	400 nM
	Rtyp23S_P	NR_076209.1: 1965–1986	6-FAM-TGCACATTT-ZEN-GACTTCTAACACC-IABkFQ	300 nM

PCR = polymerase chain reaction; rRNA = ribosomal RNA.

* Oligonucleotide modifications: ABY and 6-FAM (6-carboxyfluorescein) are fluorescent dyes; QSY, ZEN, and IABkFQ (Iowa Black Fluorescent Quencher) are nonfluorescent acceptor dyes.

### Reference tests.

A duplex *R. felis*/*R. typhi ompB* rtPCR assay was used to assess the performance of the new assay and was performed with two modifications: PerfeCTa Multiplex qPCR Supermix (Quantabio, Beverly, MA) was used as the master mix and the ABI 7500 FAST DX Sequence Detection System was used for rtPCR.^13^ A nested 23S rRNA RT-PCR sequencing assay was developed and used to resolve discrepant results between the 23S rRNA RT-rtPCR assay and the *ompB* rtPCR assay (Supplemental Materials).

### Assay performance characteristics.

Assay exclusivity was assessed using nucleic acids from 14 members of the order Rickettsiales and 28 pathogens causing fever and rash illnesses (Supplemental Table 1). Analytical sensitivity was determined for the 23S rRNA RT-rtPCR and the *ompB* rtPCR assays using quantified total nucleic acids from *R. felis* and *R. typhi* spiked into pooled nucleic acids from human whole blood or sera at concentrations of descending 10-fold increments from 1,000 to 0.01 genome copies per 5 µL. Each nucleic acid concentration was tested in replicates of five, and the limit of detection (LOD) for each analyte was defined as the lowest number of genomic copies at which all five replicates were detected. The agreement between assays for each analyte was assessed for a panel of 87 clinical specimens and 30 *R. felis*–contrived specimens. Discrepant results between assays were resolved using the nested 23S rRNA RT-PCR sequencing assay.

## RESULTS

### SNP identification and RT-rtPCR assay development.

**Figure 1.**
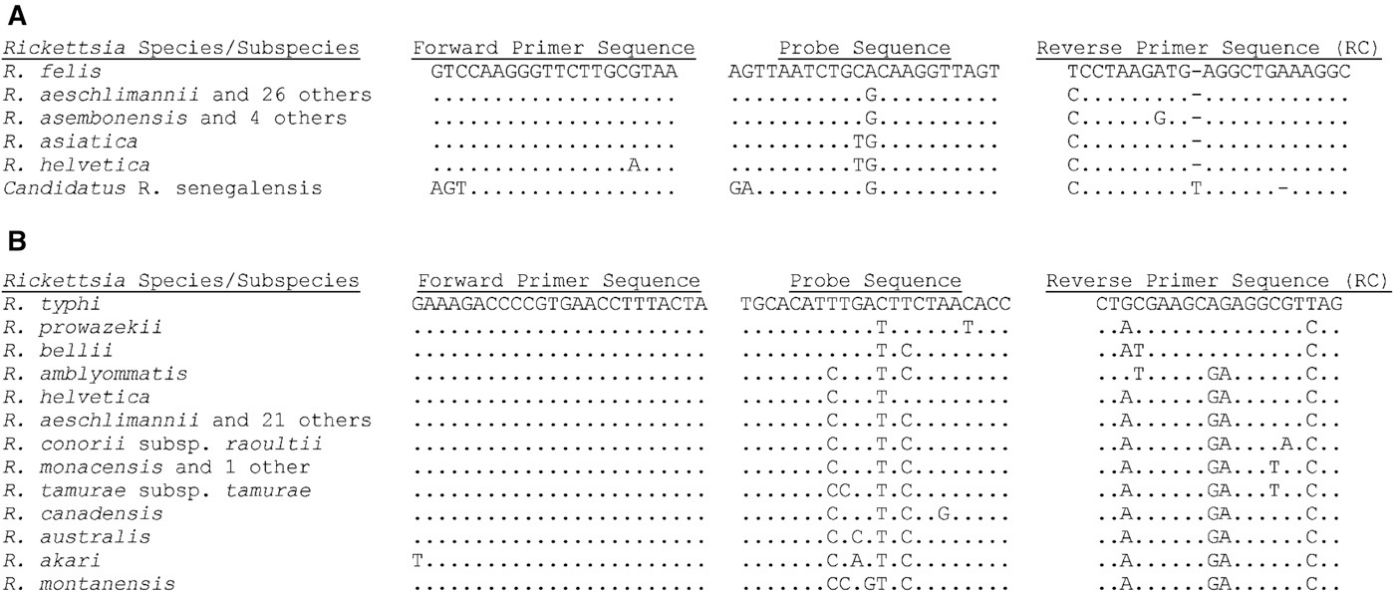
Alignment of *Rickettsia felis* (**A**) and *Rickettsia typhi* (**B**) 23S rRNA primer and probe sequences with sequences from 34 *Rickettsia* species and subspecies. The *R. felis*– and *R. typhi*–specific single nucleotide polymorphisms are located near the center of the probe sequence. Dots indicate identical nucleotide at that position. A dash indicates a nucleotide insertion/deletion. RC = reverse complement.

*Rickettsia felis*– and *R. typhi*–specific SNPs were identified through the alignment of 23S rRNA sequences from 35 *Rickettsia* species and subspecies. An *R. felis* 23S rRNA G1333A SNP and an *R. typhi* 23S rRNA T1976C SNP were selected for the design of dual-labeled allelic discrimination probes and the development of the duplex RT-rtPCR assay. The regions amplified by the RT-rtPCR assay included nucleotides 1302–1369 of the *R. felis* 23S rRNA sequence NR_076359.1 and nucleotides 1929–2020 of the *R. typhi* 23S rRNA sequence NR_076209.1 and correspond to amplicon sizes of 67 and 91 bp, respectively (Table 1). A search of NCBI databases indicated that the respective primer and probe sequences were conserved among the available 23S rRNA sequences for *R. felis* strains (URRWXCal2, Pedreira, LSU, LSU-Lb, and BBayA_MAG) and *R. typhi* strains (Wilmington, TM2540, TH1527, and B991CWPP). Alignments of the *R. felis* and *R. typhi* primer and probe sequences with sequences from 34 other *Rickettsia* species and subspecies are shown in Figure 1A and B. Although not considered a validly named species at this time, the 23S rRNA sequence from *Candidatus* Rickettsia senegalensis, a genetic near neighbor of *R. felis*, was also included in the primer and probe sequence alignment (Figure 1A). The alignments illustrate that the *R. felis* G1333A and *R. typhi* T1976C SNPs, positioned near the center of the probe sequences in Figure 1, are species specific.

### Assay exclusivity.

Assay exclusivity was assessed using a panel of nucleic acids from 14 members of the order Rickettsiales, including *Rickettsia akari*, *Rickettsia amblyommatis*, *Rickettsia asembonensis*, *Rickettsia massiliae*, *Rickettsia parkeri*, *Rickettsia* strain 364D, *Rickettsia prowazekii*, *Rickettsia rhipicephali*, *Rickettsia rickettsii*, *Rickettsia sibirica*, *Rickettsia tillamookensis*, *Anaplasma phagocytophilum*, *Ehrlichia chaffeensis*, and *Orientia tsutsugamushi*, and 28 pathogens were considered in the differential diagnosis of fever and rash illnesses, including coxsackieviruses A6 and A16, enterovirus A71, human herpesviruses 1–6, measles virus, rubella virus, human immunodeficiency virus, West Nile virus, dengue virus types 1–4, Zika virus, *Coxiella burnetii*, *Bartonella bacilliformis*, *Bartonella henselae*, *Bartonella quintana*, *Neisseria meningitidis*, *Neisseria gonorrhoeae*, *Treponema pallidum*, *Streptococcus pyogenes*, *Salmonella typhi*, and *Staphylococcus aureus*. Cross reactivity was not observed with any of the nucleic acids tested.

### Analytical sensitivity and assay agreement.

The analytical sensitivity of the 23S rRNA RT-rtPCR and *ompB* rtPCR assays was determined for the detection of total nucleic acids from the *R. felis* strain Baton Rouge and *R. typhi* strain Wilmington. Among a background of nucleic acids derived from blood or serum specimens, the LOD of the 23S rRNA RT-rtPCR assay for *R. felis* and *R. typhi* was 0.1 and 1 genomic copies per reaction, respectively, whereas the LOD of the *ompB* rtPCR assay for *R. felis* and *R. typhi* was 100 and 10 genomic copies per reaction, respectively (Tables 2–5).

**Table 2:** *Rickettsia felis* assay limit of detection comparison in blood matrix

*R. felis* Genomic Copies/Reaction	*R. felis* 23S rRNA RT-rtPCR		*R. felis ompB* rtPCR	
	Number of Replicates Detected	Mean Cycle Threshold Value*	Number of Replicates Detected	Mean Cycle Threshold Value*
1,000	5/5	22.32 (0.27)	5/5	30.97 (0.11)
100	5/5	25.56 (0.29)	5/5	34.07 (0.10)
10	5/5	28.94 (0.24)	4/5	36.85 (0.54)
1	5/5	32.31 (0.23)	1/5	38.09
0.1	5/5	35.87 (0.25)	1/5	38.14
0.01	4/5	41.15 (1.39)	0/5	Not Detected

rRNA = ribosomal RNA; RT-rtPCR = reverse transcription real-time polymerase chain reaction.

* SD shown in parentheses.

**Table 3:** *Rickettsia typhi* assay limit of detection comparison in blood matrix

*R. typhi* Genomic Copies/Reaction	*R. typhi* 23S rRNA RT-rtPCR		*R. typhi ompB* rtPCR	
	Number of Replicates Detected	Mean Cycle Threshold Value*	Number of Replicates Detected	Mean Cycle Threshold Value*
1,000	5/5	23.70 (0.17)	5/5	29.08 (0.14)
100	5/5	26.89 (0.37)	5/5	32.76 (0.28)
10	5/5	30.73 (0.27)	5/5	35.97 (0.72)
1	5/5	33.87 (1.07)	1/5	38.04
0.1	2/5	36.50 (0.71)	0/5	Not Detected
0.01	1/5	35.83	0/5	Not Detected

rRNA = ribosomal RNA; RT-rtPCR = reverse transcription real-time polymerase chain reaction.

* SD shown in parentheses.

**Table 4:** *Rickettsia felis* assay limit of detection comparison in serum matrix

*R. felis* Genomic Copies/Reaction	*R. felis* 23S rRNA RT-rtPCR		*R. felis ompB* rtPCR	
	Number of Replicates Detected	Mean Cycle Threshold Value*	Number of Replicates Detected	Mean Cycle Threshold Value*
1,000	5/5	22.98 (0.34)	5/5	31.08 (0.10)
100	5/5	26.28 (0.33)	5/5	34.49 (0.56)
10	5/5	29.56 (0.21)	3/5	37.18 (0.07)
1	5/5	32.90 (0.25)	1/5	38.99
0.1	5/5	36.86 (0.34)	1/5	39.59
0.01	2/5	40.49 (0.40)	0/5	Not Detected

rRNA = ribosomal RNA; RT-rtPCR = reverse transcription real-time polymerase chain reaction.

* SD shown in parentheses.

**Table 5:** *Rickettsia typhi* assay limit of detection comparison in serum matrix

*R. typhi* Genomic Copies/Reaction	*R. typhi* 23S rRNA RT-rtPCR		*R. typhi ompB* rtPCR	
	Number of Replicates Detected	Mean Cycle Threshold Value*	Number of Replicates Detected	Mean Cycle Threshold Value*
1,000	5/5	24.60 (0.10)	5/5	29.51 (0.25)
100	5/5	27.93 (0.25)	5/5	32.98 (0.29)
10	5/5	31.22 (0.50)	5/5	36.65 (0.72)
1	5/5	34.81 (0.89)	1/5	37.56
0.1	1/5	37.21	0/5	Not Detected
0.01	0/5	Not Detected	0/5	Not Detected

rRNA = ribosomal RNA; RT-rtPCR = reverse transcription real-time polymerase chain reaction.

* SD shown in parentheses.

The agreement between the 23S rRNA RT-rtPCR and the *ompB* rtPCR assays was assessed by testing the panel of clinical specimens from 87 individuals with suspected *Rickettsia* infections. Cases from 15 counties were represented in this study, with the majority (75.9%) collected from patients residing in Los Angeles and Orange Counties. Most case-patients (82.8%) were seropositive for *Rickettsia* as determined by a commercial or clinical laboratory prior to submission to the CDPH. Clinical information was available for 81 case-patients, of whom 49.4% reported a rash and 63% met the clinical criteria for defining suspected rickettsiosis cases for surveillance purposes.^15^ The number of days elapsed between the onset of symptoms and the date of specimen collection ranged from 1 to 17 days, with a median of 6 days. The panel was supplemented with 30 contrived *R. felis* specimens, bringing the total number of specimens tested with both assays to 117. The agreement between assays for *R. felis* and *R. typhi* detection was 93.2% and 95.7%, respectively (Tables 6 and 7).

**Table 6:** Agreement between assays for *Rickettsia felis* detection

*R. felis* Analyte	*ompB* rtPCR Detected	*ompB* rtPCR Not Detected
23S rRNA RT-rtPCR Detected	23	8
23S rRNA RT-rtPCR Not Detected	0	86

rRNA = ribosomal RNA; RT-rtPCR = reverse transcription real-time polymerase chain reaction.

**Table 7:** Agreement between assays for *Rickettsia typhi* detection

*R. typhi* Analyte	*ompB* rtPCR Detected	*ompB* rtPCR Not Detected
23S rRNA RT-rtPCR Detected	30	4
23S rRNA RT-rtPCR Not Detected	1	82

rRNA = ribosomal RNA; RT-rtPCR = reverse transcription real-time polymerase chain reaction.

*Rickettsia felis* was detected in 23 specimens, all contrived, by both the 23S rRNA RT-rtPCR and *ompB* rtPCR assays. Discrepant *R. felis* results were obtained for eight specimens (two clinical and six contrived), with all eight detected only with the 23S rRNA RT-rtPCR assay. Repeat testing of these eight specimens resulted in *R. felis* detections for one clinical and six contrived specimens with the 23S rRNA RT-rtPCR assay and for two contrived specimens with the *ompB* rtPCR assay. All six contrived specimens, but neither of the two clinical specimens, were confirmed as *R. felis* detections using the nested RT-PCR sequencing assay.

*Rickettsia typhi* was detected in 30 clinical specimens with both assays. Five specimens returned discrepant *R. typhi* results: detections in four specimens by only the 23S rRNA RT-rtPCR assay and one specimen by only the *ompB* rtPCR assay. Upon repeat testing of these five specimens, *R. typhi* was detected by both assays in three of the four specimens initially detected by only the 23S rRNA RT-rtPCR assay. *Rickettsia typhi* was not detected by either assay for the remaining two specimens. Testing of these five specimens with the nested RT-PCR sequencing assay agreed with the original 23S rRNA RT-rtPCR testing results: *R. typhi* detected in four specimens and not detected in one specimen. The overall case positivity rate for *R. typhi* detection in clinical specimens was 39.1%, with the detections ranging from 1 to 14 days after symptom onset. All positive *R. typhi* cases resided in either Los Angeles or Orange Counties.

## DISCUSSION

The presence of hundreds to thousands of rRNA molecules in a bacterial cell offers multicopy targets for the design of analytically sensitive RT-rtPCR assays for bacterial detection.^12^^,^^16^^–^^20^ Recently, Chung et al.^12^ applied ribosomal RT-rtPCR for the genus-level detection of *Rickettsia* in clinical specimens. The authors demonstrated that a 23S rRNA RT-rtPCR assay had a 100-fold higher analytical sensitivity for *Rickettsia* detection than a single-copy 50S ribosomal protein L16 gene rtPCR assay. The design of species-specific ribosomal RT-rtPCR assays can be challenging because of the lack of significant rRNA sequence divergence between species.^20^ To circumvent this shortcoming, we targeted species-specific 23S rRNA SNPs for the detection of *R. felis* and *R. typhi* by duplex RT-rtPCR. Compared with an established duplex *ompB* rtPCR assay, the 23S rRNA RT-rtPCR assay was 1,000-fold and 10-fold more analytically sensitive for the detection of *R. felis* and *R. typhi*, respectively. The superior analytical sensitivity of the 23S rRNA RT-rtPCR assay carried over to the testing of clinical specimens and *R. felis*–contrived specimens. Initial testing of samples with both assays revealed eight additional *R. felis* and four additional *R. typhi* detections with the 23S rRNA RT-rtPCR assay. The six contrived *R. felis* and all four of the clinical *R. typhi* detections were confirmed by a nested RT-PCR sequencing assay. However, two *R. felis* clinical specimen detections with the 23S rRNA RT-rtPCR assay could not be confirmed because either the results were falsely positive or the analyte concentrations were at or beyond the lower LOD for the resolver test.

In addition to superior analytical sensitivity, in silico analysis of *Rickettsia* 23S rRNA sequences and exclusivity testing indicated that the RT-rtPCR assay is highly specific for *R. felis* and *R. typhi*. Several *R. felis*–like organisms recently have been described and the genomes sequenced, including *R. asembonensis*, *Rickettsia hoogstraalii*, and *Candidatus* R. senegalensis.^21^^–^^23^ All three of these *Rickettsia* have an alternate SNP allele at nucleotide 1333 of the *R. felis* 23S rRNA target sequence and are predicted to be nonreactive in the 23S rRNA RT-rtPCR assay. Indeed, *R. asembonensis* was included in the assay exclusivity panel and was found to be nonreactive. In contrast, the *R. felis* genetic near neighbors *R. asembonensis* and *Candidatus* R. senegalensis shared sequence identity with the *R. felis ompB* rtPCR target, reducing the specificity of this assay for *R. felis* detection.

The number of FBT cases recorded annually by Texas and California has increased in the last 10 years.^2^^,^^3^ Texas has also noted a geographic expansion in case distribution, whereas reported cases in California remain mostly restricted to Los Angeles and Orange Counties, with 90% of the cases acquired in a suburban setting.^24^^,^^25^ In this environment, the transmission of flea-borne rickettsioses to humans likely is enabled by opossums serving as the reservoir host and cat fleas functioning as the vector. Field studies conducted in regions of southern California with endemic foci of flea-borne rickettsioses have found that cat flea infections with *R. felis* are much more prevalent than infections with *R. typhi* and have led to the proposal that *R. felis* may be the principal cause of flea-borne rickettsioses.^7^^,^^9^ Our study counters this hypothesis by demonstrating that 39.1% of the suspected flea-borne rickettsiosis cases were detected as *R. typhi* infections, whereas only 0–2.3% of the cases were detected as *R. felis* infections; our data support the counter proposal that most California cases are caused by *R. typhi*.^26^

There are two limitations to our study. First, the study was geographically restricted to California and does not account for strain variation that may occur in other parts of the world. In addition, very few *R. felis* and *R. typhi* 23S rRNA sequences are available in public databases to assess sequence variation by in silico analyses. Further evaluation of the assay with geographically diverse *R. felis* and *R. typhi* samples and expansion of *Rickettsia* 23S rRNA sequences in public databases are warranted. Second, most specimens in this study were serum samples. Although frequently unavailable because of lags in case reporting, the case positivity rate may have benefitted from the use of more productive specimen types such as whole blood and, optimally, skin biopsies of rash lesions.^27^^–^^30^ However, even for these specimen types, the detection of *Rickettsia* can be challenging owing to transient bacteremia and diurnal fluctuations in bacterial loads for blood specimens and the variable presentation of a rash for the collection of skin biopsies.^1^^,^^31^ Nonetheless, the RT-rtPCR assay targeting multiple copies of 23S rRNA offered a significant advantage over rtPCR assays targeting single-copy DNA sequences and promises to provide a powerful new surveillance tool for detecting *R. felis* and *R. typhi* cases.

We have described the development of an improved duplex molecular diagnostic test for the detection of flea-borne rickettsioses. The assay demonstrated enhanced analytical sensitivity and specificity for *R. felis* and *R. typhi* detection relative to an established duplex rtPCR assay. Testing of surveillance specimens collected over the last 6 years with these two assays demonstrated that *R. typhi* is the predominant cause of flea-borne rickettsioses in California and confirmed that FBT is largely restricted to Los Angeles and Orange Counties. The implementation of this rapid, analytically sensitive, and accurate test will facilitate public health surveillance efforts to monitor flea-borne rickettsiosis trends, identify outbreaks and epicenters of disease transmission, and help guide targeted intervention to reduce infection rates.

## Supplemental Materials

10.4269/ajtmh.23-0884 Supplemental Materials
