# Supplementary material for: Duplex Reverse-Transcription Real-Time Polymerase Chain Reaction Assay Targeting 23S rRNA Single Nucleotide Polymorphisms for the Detection of Flea-Borne Rickettsioses
Source: Am J Trop Med Hyg. 2024 Jul 16;111(3):569–74. doi: 10.4269/ajtmh.23-0884 (PMC11376180; doi:10.4269/ajtmh.23-0884)
Supplement: Supplemental Materials [file tpmd230884.SD1.pdf]

Supplemental Table 1. Exclusivity panel of nucleic acids

Organism	Strain	Source
Rickettsia species	364D	CDC
Rickettsia akari	Kaplan	CDC
R. amblyommatis	GAT-30V	CDC
R. asembonensis	NMRC11	CDC
R. massiliae	AZT80	CDC
R. parkeri	Maculatum 20	CDC
R. prowazekii	Madrid II	CDC
R. rhipicephali	20-4164	CDC
R. rickettsii	Sheila Smith	CDC
R. sibirica	246	BEI Resources
R. tillamookensis	Tillamook 23	CDC
Anaplasma phagocytophilum	NCH-1	BEI Resources
Erhlichia chaffeensis	St. Vincent	BEI Resources
Orientia tsutsugamushi	Karp	CDC
Coxsackievirus A6	05871	CDPH
Coxsackievirus A16	02550	CDPH
Enterovirus A71	03669	CDPH
Dengue virus type 1	HAW	CDPH
Dengue virus type 2	NGC	CDPH
Dengue virus type 3	H87	CDPH
Dengue virus type 4	H41	CDPH

Herpes herpesvirus 1	019354	CDPH
Herpes herpesvirus 2	020806	CDPH
Human herpesvirus 3	Batson	CDPH
Human herpesvirus 4	B95-8	CDPH
Human herpesvirus 5	TC-844	CDPH
Human herpesvirus 6	Z-29	CDPH
Human immunodeficiency virus type 1	23135	CDPH
Measles virus	04850	CDPH
Rubella virus	00182	CDPH
West Nile virus	TC 86300	CDPH
Zika virus	PRVABC59	CDPH
Bartonella bacilliformis	ATCC 35685	CDPH
Bartonella henselae	ATCC 49882	CDPH
Bartonella quintana	ATCC 51694	CDPH
Coxiella burnetii	Nine mile	CDPH
Neisseria meningitidis	M11095	CDPH
Neisseria gonorrhea	WHO_Z	CDPH
Treponema pallidum	A3-2	CDPH
Streptococcus pyogenes	ATCC 21059	CDPH
Salmonella typhi	80-2002	CDPH
Staphylococcous aureus	AR_0225	CDPH

CDC = Centers for Disease Control and Prevention; CDPH = California Department of Public Health

Supplemental Table 2. *Rickettsia* species and subspecies sequences used for 23S rRNA analyses

Species/Subspecies	Strain	GenBank Accession Number
R. aeschlimannii	MC16	CCER01000011.1
R. africae	ESF-5	CP001612.1
R. akari	Hartford	CP000847.1
R. amblyommatis	GAT-30V	CP003334.1
R. asembonensis	Perak	CP116496.1
R. asiatica	Maytaro1284	AP019563.1
R. australis	Cutlack	CP003338.1
R. bellii	RML369-C	CP000087.1
R. canadensis	CA410	CP003304.1
R. conorii subsp. conorii	Malish 7	AE006914.1
R. conorii subsp. indica	ITTR	AJHC01000003.1
R. conorii subsp. israelensis	ISTT CDC1	AJVP01000009.1
R. conorii subsp. raoultii	Khabarovsk	CP010969.1
R. felis	URRWXCal2	NR_076359.1
R. fournieri	AUS118	OFAL01000006.1
R. gravesii	BWI1	AWXL01000005.1
R. heilongjiangensis	054	CP002912.1
R. helvetica	C9P9	AICO01000001.1
R. honei	RB	AJTT01000002.1
R. hoogstraalii	Croatica	CCXM01000001.1
R. japonica	YH	AP011533.1

R. massiliae	MTU5	CP000683.1
R. monacensis	IrR/Munich	LN794217.1
R. montanensis	OSU 85-930	CP003340.1
R. parkeri	Portsmouth	CP003341.1
R. peacockii	Rustic	CP001227.1
R. prowazekii	Breinl	CP004889.1
R. rhipicephali	3-7-female6-CWPP	CP003342.1
R. rickettsii	Iowa	CP000766.3
R. sibirica	246	AABW01000001.1
R. slovaca	D-CWPP	CP003375.1
R. tamurae subsp. buchneri	PalLab	CP113531.1
R. tamurae subsp. tamurae	AT-1	CCMG01000015.1
R. tillamookensis	Tillamook 23	CP060138.2
R. typhi	Wilmington	NR_076209.1

Nested 23S rRNA RT-PCR Sequencing Reference Test

A nested 23S rRNA RT-PCR sequencing assay was developed and used to resolve discrepant results between the 23S rRNA RT-rtPCR assay and the *ompB* rtPCR assay. The outer RT-PCR was performed using the PrimeScript One-Step PCR kit (Takara Bio) in a 50 µL reaction volume consisting of 10 µL of nucleic acids and primers Rk_23S_1137F (5'-GGCTCAAGTCATGTACCGAA-3') and Rk_23S_1609R (5'-ACAGGTCATCTTTCTTCCGA-3') each at a concentration of 600 nM. RT-PCR was performed using a T-100 thermal cycler (Bio-Rad Laboratories, Hercules, CA) with cycling parameters consisting of 50°C for 30 min, 94°C for 2 min, 50 cycles at 94°C for 30 sec, 52°C for 30 sec, and 72°C for 1 min, and a final hold at 72°C for 2 min. The inner PCR was conducted in a reaction volume of 25 µL using 1X Q5 Hot Start, High Fidelity master mix (New England Biolabs, Ipswich, MA), primers Rk_23S_1143 (5'- AGTCATGTACCGAAGATGCG-3') and Rk_23S_1606R (5'- GGTCATCTTTCTTCCGAAGTTAC-3) each at a concentration of 300 nM, and 1 µL of the outer RT-PCR product. Amplification was performed in a T-100 thermal cycler using cycling parameters consisting of 98°C for 30 sec, 35 cycles at 98°C for 5 sec, 61°C for 20 sec, and 72°C for 30 sec, and a hold at 72°C for 2 min. An expected amplified product size of 464 bp was assessed using the D1000 ScreenTape system and TapeStation 4200 instrument (Agilent Technologies, Santa Clara, CA). Bidirectional Sanger sequencing using primers Rk_23S_1143F and Rk_23S_1606R was performed by a commercial laboratory (Sequetech Corporation, Mountain View, CA). Sequences were edited and assembled with Geneious v10 software (Biomatters Inc., Boston, MA) and a BLAST search of the NCBI nr/nt database performed for *Rickettsia* species identification.
